# Editor’s introduction

**DOI:** 10.1186/s40854-022-00416-8

**Published:** 2022-11-15

**Authors:** Gang Kou

**Affiliations:** Southwestern University of Finance and Economics, Chengdu, China

The 35th issue of Financial Innovation (FIN), Volume 8, No.5 (2022) presents 19 papers contributed by authors and co-authors from sixteen countries and areas: Australia, Brazil, Canada, China, Hong Kong, Iran, Italy, Japan, Korea, Netherlands, Saudi Arabia, South Africa, Taiwan, Türkiye, Tunisia and USA.

- Fintech (7 papers)

The paper “Exploring biometric identification in FinTech applications based on the modified TAM” constructs a multi-object framework to evaluate biometrics in FinTech applications and helps readers understand how users view biometrics in FinTech applications.

The paper “Fintech, regtech, and financial development: evidence from China” investigates the influence of fintech on developments in China’s financial sector across 290 cities and 31 provinces and suggests a policy framework for balanced fintech sector growth in developing countries.

The paper “Predicting cash holdings using supervised machine learning algorithms” implies that with more advanced algorithms, considerable improvements are observed with a maximum of 42% improvement in RMSE values. The XGBoost algorithm yielded the best results.

The paper “How does a data strategy enable customer value? The case of FinTechs and traditional banks under the open finance framework” reveals that there is a form of external information asymmetry between the customer and the bank, and also an internal asymmetry between bank departments, as their visibility on information about the same customer may differ.

The paper “An overview of Fintech applications to solve the puzzle of health care funding: state-of-the-art in medical crowdfunding” describes crowdfunding’s relations with the health care industry, seeking the crowdfunding levers and the effects that crowdfunding has on the innovation and accessibility of medical treatments.

The paper “An algorithm for the anchor points of the PPS of the BCC model” proposes an approach based on a variant of super-efficiency models and their duals. The necessary and sufficient conditions for the characterization of the anchor points are also provided. Finally, the applicability of the proposed model is illustrated with some numerical examples.

The paper “The social representation of Fintech from the perspective of traditional financial sector professionals: evidence from Brazil” compares its findings with the scientific literature on the concept of fintech. It emphasizes the need to develop a legal and regulatory framework for the performance of fintech in emerging markets.

- Financial risk management and analysis (5 papers)

The paper “Impact of COVID-19 on G20 countries: analysis of economic recession using data mining approaches” offers some crucial recommendations to handle pandemics in terms of the suggested economic systems by identifying the challenges that the G20 countries have experienced.

The paper “Does supplier concentration matter to investors during the COVID-19 crisis: evidence from China?” finds that the concentration risk of suppliers is a significant consideration for China stock market investors, especially under the potential financial distress at the firm level induced by the COVID-19 crisis.

The paper “Contingent convertible lease modeling and credit risk management” investigates three types of contingent leases and determines a lease agreement to finance an investment project and a solution for managing credit risk.

The paper “Clues from Networks: Quantifying Relational Risk for Credit Risk Evaluation of SMEs” reveals that relational risk score significantly improves both discrimination and granting performances of credit risk evaluation of SMEs, providing valuable managerial and practical implications for financial institutions.

The paper “'Smart' copycat mutual funds: On the performance of partial imitation strategies” concludes that smart copycatting is another skill of successful fund managers, which is contrary to previous research.

- Financial development (2 papers)

The paper “Exploring the moderating role of financial development in environmental Kuznets curve for South Africa: fresh evidence from the novel dynamic ARDL simulations approach” provides some crucial policy recommendations and fresh perspectives for South Africa as it develops national initiatives to support ecological sustainability and reach its net zero emissions goal.

The paper “Effects of Financial Development and Capital Accumulation on Labor Productivity in Sub-Saharan Africa: New insight from Cross Sectional Autoregressive Lag Approach” finds that financial progress in the region over time leads to an increase in productivity of labor and also the accumulation of capital. Furthermore, financial markets have a progressive impact on the productivity of labor within sub-Saharan African regions.

- Cryptocurrency (5 papers)

The paper “Robust estimation of time-dependent precision matrix with application to the cryptocurrency market” applies the methodology to synthetic data to test its performances. The proposed method is remarkably suited for the considered application because the cryptocurrency market is characterized by a peculiar time-dependent and very turbulent nature.

The paper “Manipulation of the Bitcoin market: an agent-based study” suggests that the presence of the fraudulent agent was essential to obtain Bitcoin price development in the given time period and provides important insights to further the understanding of exchange manipulation with possible impacts on the entire market.

The paper “Consumer choices under new payment methods” suggests a payment portfolio model that includes new payment methods that have emerged from the development of cryptocurrency markets and central bank digital currencies (CBDCs). It concludes that the higher the government’s interest rate on CBDCs, the more consumers will use CBDCs than deposits.

The paper “An analysis of the acquisition of a monetary function by cryptocurrency using a multi-agent simulation model” finds that cryptocurrencies can function as a second currency when 20% of all agents accept it for payment and when 50% of agents are aware of this fact.

The paper “Automated market makers and decentralized exchanges: a DeFi primer” provides an intuitive geometric representation of how an Automated Market Makers (AMMs) operates, and a clear delineation of the similarities and differences across the various types of AMMs.

